# Irinotecan plus folinic acid/continuous 5-fluorouracil as simplified bimonthly FOLFIRI regimen for first-line therapy of metastatic colorectal cancer

**DOI:** 10.1186/1471-2407-4-38

**Published:** 2004-07-20

**Authors:** Andreas Teufel, Silke Steinmann, Jürgen Siebler, Christiane Zanke, Herbert Hohl, Bernd Adami, M Schroeder, O Klein, Thomas Höhler, Peter R Galle, Michael Heike, Markus Moehler

**Affiliations:** 1 Dept. of Internal Medicine I, Outpatient Unit for GI Cancer, Johannes Gutenberg University, Mainz, Germany; 2 Hospital Bad Ems, Outpatient Unit, Bad Ems, Germany; 3 General practice, Alzey, Germany; 4 General practice, Landau, Germany; 5 General practice, Kostheim, Germany

**Keywords:** irinotecan, 5-fluorouracil, safety, metastatic colorectal cancer, neoadjuvant

## Abstract

**Background:**

Combination therapy of irinotecan, folinic acid (FA) and 5-fluorouracil (5-FU) has been proven to be highly effective for the treatment of metastatic colorectal cancer. However, in light of safety and efficacy concerns, the best combination regimen for first-line therapy still needs to be defined. The current study reports on the bimonthly FOLFIRI protocol consisting of irinotecan with continuous FA/5-FU in five German outpatient clinics, with emphasis on the safety and efficiency, quality of life, management of delayed diarrhea, and secondary resection of regressive liver metastases.

**Methods:**

A total of 35 patients were treated for metastatic colorectal cancer. All patients received first-line treatment according to the FOLFIRI regimen, consisting of irinotecan (180 mg/m^2^), L-FA (200 mg/m^2^) and 5-FU bolus (400 mg/m^2^) on day 1, followed by a 46-h continuous infusion 5-FU (2400 mg/m^2^). One cycle contained three fortnightly administrations. Staging was performed after 2 cycles. Dosage was reduced at any time if toxicity NCI CTC grade III/IV was observed. Chemotherapy was administered only to diarrhea-free patients.

**Results:**

The FOLFIRI regimen was generally well tolerated. It was postponed for one-week in 51 of 415 applications (12.3%). Dose reduction was necessary in ten patients. Grade III/IV toxicity was rare, with diarrhea (14%), nausea/vomiting (12%), leucopenia (3%), neutropenia (9%) and mucositis (3%). The overall response rate was 31% (4 CR and 7 PR), with disease control in 74%. After primary chemotherapy, resection of liver metastases was achieved in three patients. In one patient, the CR was confirmed pathologically. Median progression-free and overall survival were seven and 17 months, respectively.

**Conclusions:**

The FOLFIRI regimen proved to be safe and efficient. Outpatient treatment was well tolerated. Since downstaging was possible, combinations of irinotecan and continuous FA/5-FU should further be investigated in neoadjuvant protocols.

## Background

Patients with advanced *C*olo*R*ectal *C*ancer (CRC) have been demonstrated to benefit from chemotherapeutic treatment in terms of both quality and duration of life [1]. Of these treatments, fluoropyrimidines are the most often used and best investigated drugs [2,3]. 5-Fluorouracil (5-FU)-based chemotherapy, – usually biomodulated with folinic acid (FA) to increase its affinity for thymidylate synthase [4] -, was associated with an approximate doubling of the median survival compared to routine standard of care [5]. Additionally, recent phase III studies suggested that combinations of FA/5-FU with irinotecan or oxaliplatin resulted in improved response rates and prolonged survival [6-9]. These encouraging results prompted the use of combination therapy of irinotecan and FA/5-FU as a first line chemotherapy in CRC.

Irinotecan (CPT-11), a potent inhibitor of the enzyme topoisomerase I, has demonstrated anti-tumorogenic activity in metastatic colorectal cancer, when used alone or in combinations with FA/5-FU, as adjuvant or palliative treatment (for review see 10 or 11). In randomized phase III clinical trials, second-line therapy with irinotecan significantly improved survival compared to supportive care [12] or to infusional FA/5-FU [13]. In the first-line setting of metastatic colorectal cancer, two randomized multi-center phase III clinical trials demonstrated synergistic activity of irinotecan with both bolus and infusional FA/5-FU regimens [6,7]. In both studies, combinations of irinotecan and FA/5-FU were superior to the control arms, irinotecan alone or FA/5-FU, specifically in regard to response rate, progression-free, and overall survival.

However, the best regimen of irinotecan and FA/5-FU has yet to be defined. Altogether, irinotecan combined with continuous FA/5-FU infusions seemed to be superiorly active and less toxic than combination with FA/5-FU bolus regimens [6,7]. Recently, irinotecan was investigated in a bi-monthly protocol with bolus FA/5-FU and a continuous 48 h infusion 5-FU (simplified LV5-FU2 regimen; FOLFIRI) [14-16]. In a consecutive phase III clinical trial, a response rate of 56% was achieved with FOLFIRI, as compared to 39% achieved with the original Saltz protocol [6,14].

Furthermore, irinotecan schedules of weekly and of once every two or three weeks demonstrated similar efficacy and quality of life, as well as significantly lower incidences of severe diarrhea in patients with 5-FU-refractory, metastatic colorectal cancer [15-17]. In contrast to the irinotecan and bolus FA/5-FU regimen, which has attracted criticism due to unexpectedly high early death rates due to gastrointestinal toxicity and thromboembolic events observed in two subsequent trials [18,19], no increased 60-day mortality rate was found in two recent trials, each with continuous 5-FU treatment arms [14,17,20,21].

To date, little data is available regarding irinotecan combined with the simplified bi-monthly LV5-FU2 regimen as a first-line treatment in patients with metastatic colorectal cancer [14]. Therefore, we initiated this prospective open-label, multi-center phase IV clinical trial to evaluate its toxicity and efficacy in German outpatient clinics. We were especially interested in the safety of this regimen in an outpatient setting, with particular emphasis placed on the prompt and aggressive management of delayed diarrhea with loperamide, hospitalization and parenteral rehydration in case of refractory diarrhea lasting more than 48 hours [22,23]. Furthermore, all patients were closely monitored for the possibility of resection of liver metastases after successful response.

## Methods

### Accrual and eligibility

After approval by the local ethical committee, patients were consecutively recruited from five German outpatient clinics (one university hospital, one community hospital, and three general practices). Eligibility requirements included (1) histologically documented adenocarcinoma of the colon or rectum and progressive measurable metastatic disease, (2) minimum life expectancy of three months, (3) Karnofsky performance status ≥ 60, (4) adequate hematologic, hepatic, and renal function, and (4) no prior chemotherapy for metastatic disease. Participants needed to be between 18 and 75 years of age. This study required that previous adjuvant 5-FU-based therapy with or without radiation therapy be completed at least 6 months prior to entry. Patients with CNS metastases, bowel obstruction, or ileus were excluded from the study. The study was approved by the ethics commission board responsible for all participating institutions. Prior to treatment, all patients gave written informed consent.

### Treatment and management of toxicity

As previously described [14-16], treatment consisted of the bi-monthly combination of irinotecan 180 mg/qm given as a 90-min intravenous infusion, day (d)1, FA 200 mg/m^2^ d1, 5-FU bolus 400 mg/m^2^ d1, followed by 5-FU 46 h continuous infusion 2400 mg/m^2^ (simplified LV5-FU2 schedule). To prevent expected toxicities, patients were carefully informed of the potential risk of delayed diarrhea and neutropenia and the need for early intervention with loperamide [22] and metoclopramide, prophylactic antibiotics, or hospitalization and parenteral rehydration in case of refractory diarrhea lasting more than 48 hours. Patients with loperamide-resistant diarrhea defined as loose stools persisting for more than 24 hours despite adequate treatment with loperamide would receive a trial of the oral steroid budesonide (9 mg per day for a maximum of 4 days) [23]. Atropine was given for irinotecan-related cholinergic symptoms if needed [25]. Antiemetic treatment was performed using metoclopramide or HT5 antagonists in a sequential manor. The prophylactic use of colony-stimulating factors was not permitted. Treatment was continued until one of the following occurred: disease progression, unacceptable adverse effects, or withdrawal of consent by the patient.

### Assessments

Primary measures of the study were the overall objective response rate (ORR, complete and partial responses), overall survival, and quality of life. Secondary measures included the disease control rate (ORR + stable disease), time to progression, and frequency and severity of toxicities. Quality of life was assessed after inclusion into the study and as often as possible during the course of treatment, using the EORTC QLQ-C30 (version 2) questionnaire [24].

Safety assessments and complete blood counts were performed weekly. Toxicity was graded according to National Cancer Institute Common Toxicity Criteria (NCI CTC). Toxicities not defined by NCI CTC criteria were classified as grade 0 (none), grade I (minor), grade II (moderate), grade III (severe), and grade IV (life-threatening). In case of any toxicity grade II, with the exception of hand-foot syndrome or alopecia, the next scheduled doses of irinotecan, folinic acid and 5-FU were delayed for a maximum of 1 week (or resolution of diarrhea for at least five days). In case of toxicity grade III/IV or if improvement from grade II to I (or resolution of diarrhea) was not achieved by two weeks, the following chemotherapy doses were reduced by 20 percent. If grade III/IV toxicity did not improve by 2 weeks, treatment was discontinued. Dose reductions were mandatory from the first cycle of chemotherapy in case of toxicity higher than grade II, and chemotherapy was resumed only after complete recovery from diarrhea.

Tumor response was assessed according to World Health Organization (WHO) criteria. Tumor reassessment by the same imaging method used to establish baseline tumor measurement was generally performed after every two courses of therapy until progression. A complete response (CR) was defined as complete disappearance of evidence of cancer. A partial response (PR) was defined as a reduction in the sum of the products of the bi-perpendicular diameters of all measurable lesions by at least 50%. Progressive disease (PD) was defined as an increase in the sum of the products of the greatest bi-perpendicular diameters of all measurable lesions by at least 25% or the appearance of new lesions. Stable disease was defined as any reduction or increase in measurable lesions which did not meet the criteria for PR or PD. Confirmed objective responses were those for which a follow-up scan obtained at least four weeks later demonstrated the persistence of the response. The assessment of response and progression was based on investigator-reported measurements.

### Statistical analysis

Statistical analysis including survival analysis according to Kaplan-Meier was performed with the SPSS software package. The deadline for data evaluation was March 15, 2004. Survival was measured from the time of diagnosis to the date of death or last follow-up. Progression-free survival was calculated from treatment onset to the time of progression, study withdrawal or death of any cause. Patients who received at least one dose of the treatment regimen were evaluated for toxicity, and patients who completed at least two chemotherapy cycles were evaluated for response.

## Results

Between 10/2001 and 5/2003, 35 consecutive patients (25 male, 10 female) with metastatic colorectal cancer were enrolled into the study. The median age of these patients was 62 years, ranging from 38 to 73. Baseline characteristics of all patients are summarized in table 1. Most patients were in good overall physical condition, although 60% had at least two metastatic sites. All patients had undergone surgery prior to chemotherapeutic treatment. Six patients previously had received adjuvant chemotherapy, one of them in combination with radiation therapy. All patients were evaluated for toxicity, for response, and survival.

The 35 patients received a total of 151 chemotherapy cycles (mean 4,3 per patient), consisting of 451 administrations. Overall, 51 (12,3 %) of all administrations had to be delayed for one week. During the complete study period, 19 patients had a delay of therapy for a median of nine days and in 10 patients (29%) a dose reduction was necessary at some point during the treatment period. The most common cause for discontinuation of study treatment was disease progression (18 patients, 51%). In case of discontinuation, 16 (46%) patients received a second line treatment with either oxaliplatin plus a FA/5-FU consisting regimen or an epidermal growth factor receptor antagonist.

Hematologic toxicity was mild to moderate in the majority of patients (table 2). Only one patient (3 %) had a grade III/IV leucopenia, three patients (9 %) had a grade III neutropenia, and grade III or IV anemia or thrombocytopenia were not observed. The predominant non-hematologic toxicities were nausea/vomiting and delayed diarrhea, which affected a total of 21 (60%) and 10 (29%) patients, respectively. However, grade III/IV of these side effects were only observed in 4 (11%) and 2 (6%) patients, respectively (table 3). Other non-hematological toxicities were predominantly mild, including mucositis (I°, 5 patients, 14%), fever (I°/II°, 5 patients, 14%), cholinergic syndrome (I°, 2 patients, 6%), constipation (I°, 8 patients, 23%), alopecia (I°, 9 patients, 26%, II°, 1 patient, 3%), asthenia (I°, 2 patients, 6%). Regarding the irinotecan induced delayed diarrhea, 11 patients received at least one course of loperamide [22] and 1 patient received budesonide for loperamide refractory diarrhea [23]. In two patients treatment-related hospital admissions as a result of III/IV leucopoenia and grade III diarrhea were required. Other adverse events in three patients included a bowel obstruction due to local recurrence, unexplained vertigo, and renal failure due to urethral obstruction. Pulmonary embolism did not occur in any patients during treatment.

**Figure 1:**
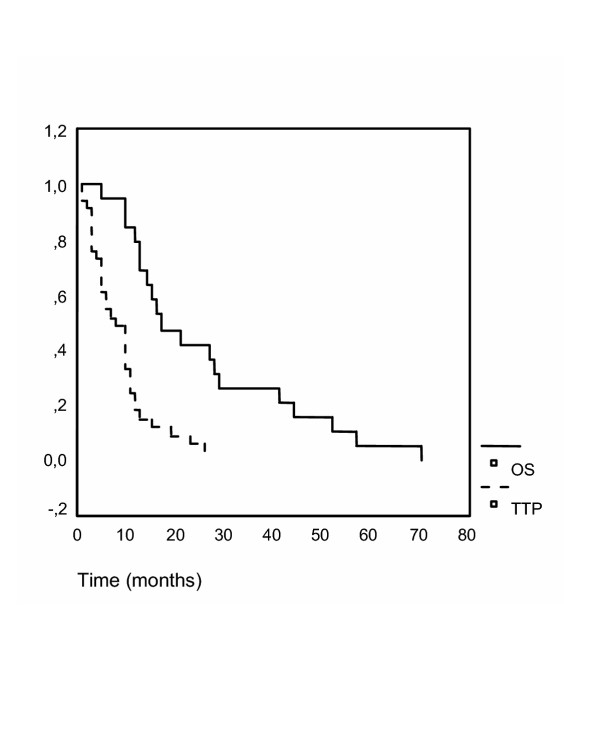
Overall survival. The time to progression (TTP) and overall survival (OS, in months) are plotted as Kaplan-Meier-curves. Overall survival is drawn as a continuous line, total progression free survival as a dotted curve. TTP and OS were 7 and 17 months, respectively.

With regard to response, four complete (CR) and seven partial responses were seen, and thus an overall response rate of 31% was observed (table 4). In addition, 15 patients (43%) had stable disease (disease control rate, 74%). Disease progression occurred in nine patients (26%). Resectability of metastases was achieved in three patients. In one patient CR, was pathologically confirmed. Median progression-free survival was seven months and overall survival was 17 months (95% confidence intervall: 9–25 months, figure 1).

**Figure 2:**
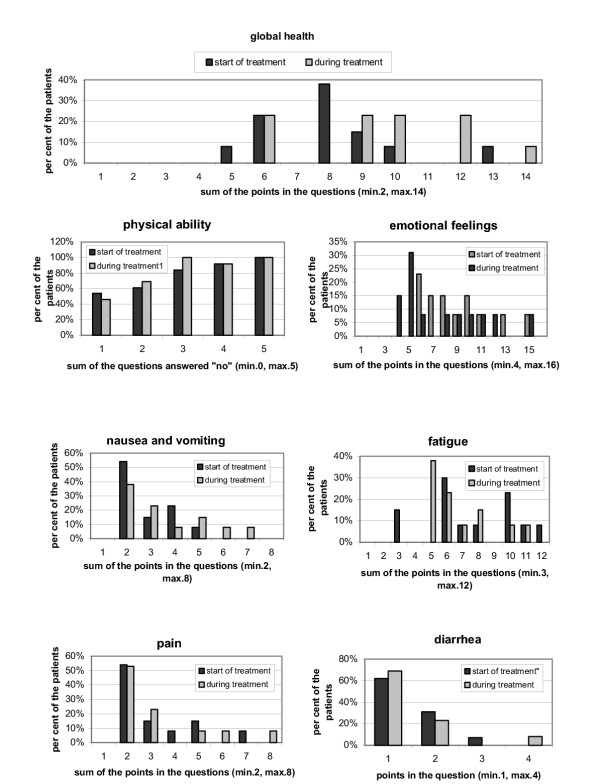
Quality of life assessed by the QLQ-C30 questionnaire. Quality of life was assessed immediately after inclusion into the study and at least once during the course of treatment, using the standardized EORTC QLQ-C30 questionnaire, version 2. Overall scores range from 0 to 100, divided into several subsets such as physical ability, emotional feelings, nausea/vomiting, fatigue, pain or diarrhea. Higher scores on global health status and physical functioning and lower scores on symptom scales and emotional assessment indicate a better quality of life.

Quality of life data were obtained before and at least once during treatment from 13 patients [24]. The 13 patients evaluated for quality of life did not differ in their pattern of response to chemotherapy from the total population of all evaluated patients. Global health status improved slightly during treatment compared to pre-therapy values (figure 2). In addition, patients treated with the FOLFIRI regimen had a small increase in emotional and physical wellbeing compared to a previously reported cohort of untreated patients. No remarkable changes in the other items of the questionaire were seen during treatment, especially with regard to therapy-dependent symptoms such as nausea and vomiting, diarrhea and pain. Slightly increased nausea and fatigue were observed in our patients. However, a clear trend could not be concluded from our data.

## Discussion

In the current phase IV study we evaluated toxicity and efficacy of the FOLFIRI combination of irinotecan with FA/5-FU as first-line chemotherapy for metastatic colorectal cancer. This combination was established within a phase I clinical trial with a recommended irinotecan dose of 180 mg/qm [15,16]. Irinotecan-containing regimens have been the most commonly used chemotherapy protocols for metastatic colorectal cancer in North America since the publications of Saltz et al. [3,6,7,12,17]. After unexpectedly high early death rates, due to gastrointestinal toxicity and thromboembolic events, which were reported in two subsequent trials due to gastrointestinal toxicity and thromboembolic events, the safety of the Saltz regimen became a subject of considerable debate [18,19]. However, in our previous experience with this regimen we did not observe any major complications [25]. Moreover, additional comprehensive data showed that combinations of irinotecan with continuous FA/5-FU, either weekly or bi-monthly, resulted in higher response rates and better survival. Therefore, we investigated the bi-monthly FOLFIRI schedule in an outpatient setting for its safety and clinical efficacy.

In the majority of our patients the FOLFIRI regimen was well tolerated. Gastrointestinal toxicity or thrombembolic events were never fatal. Most hospitalizations were for prevention rather than treatment of life-threatening conditions. Delayed diarrhea, a well known side effect of irinotecan [22], was generally managed in the outpatient setting using loperamide, which was administered to approximately one third of the patients. Budesonide, which has demonstrated activity in loperamide-refractory diarrhea was required in only one of our patients (3%).

Overall, we observed relatively low toxicity in our study, with NCI CTC grade III leucopenia amounting to 3%, diarrhea to 14% and nausea/vomiting to 11%. The toxicity observed in our study was lower than that reported by Douillard et al. in the pivotal European first-line trial in the patient group receiving weekly irinotecan (80 mg/qm), 24-h HD-5-FU (2300 mg/qm) preceded by 2-h FA 500 mg/qm [7]. In this patient group, grade III/IV toxicities were reported for leucopenia 20.4%, diarrhea 44.4% and nausea 7.4%. The lower toxicity in our study might be due to the lower per day doses of 5-FU (2400 mg/qm administered over 48 h) and L-FA (200 mg/qm) used. In the EORTC phase III study 40986, comparing first-line AIO schedule alone with irinotecan (80 mg/qm), FA 500 mg/qm and continuous FA/5-FU (2300 mg/qm), the 5-FU-dose had to be reduced to 2000 mg/qm because of initially high toxicity in an interim analysis [26]. In addition, lower toxicity in our study may have been more limited because of the early and rigorous dose reductions according to our protocol. Furthermore, we observed improved physical and emotional status and an increase in global health status during treatment [24]. This is in concordance with our previously reported data [25]. Tournigand et al. demonstrated an increase in weight of at least 5% in 35% and an improved physical status in 35% of the irinotecan/FA/5-FU treated patients, respectively [14]. Koehne et al. reported a significantly better quality of life in the irinotecan/FA/5-FU group compared to FA/5-FU [26].

The response rate achieved in our study (31%) was quite comperable tp previously published data [6,7,14,26]. In these studies, response rates were 40–56 % with time to progression (TTP) of 6–8 months. Tumor control (CR+PR+SD) was achieved in 74% of our patients, which is similar to other reports. Median progression-free and overall survival, was comparable, but slighty less than 8,5 and 21,5 months reported by Tournigand [14].

Three reasons may account for these differences in survival between the studies. The most important reason may be that a higher percentage of patients (21 patients, 60% compared to 41% and 10% reported by Tournigand and Saltz, respectively) in our study had two or more metastatic sites, indicating a larger tumor burden and consequently a worse prognosis regarding survival [6,14]. Second, our study included patients with carcinoma of the rectum (12 patients, 34% versus 15 %). And finally, as much as five (14%) of our patients had previously received adjuvant FA/5-FU-containing chemotherapy or radiotherapy compared with 10% of the patients in the other study [6].

It appears particularly noteworthy that after chemotherapy three of our patients achieved surgical resectability of their metastases. To our knowledge these results are the first ever reported to suggest a potential role for the FOLFIRI regimen in the neoadjuvant setting. Thus far, studies of regional chemotherapy for initially unresectable colorectal liver metastases could demonstrate some success with secondary curative surgery. In two recently published retrospective studies chronomodulated chemotherapy with oxaliplatin and FA/5-FU was used as neoadjuvant treatment for patients with unresectable colorectal liver metastases [27,28]. Therefore, combination regimens of irinotecan or oxaliplatin with FA/5-FU should be strongly considered as standard first-line chemotherapy for metastatic colorectal cancer. Through multidisciplinary efforts involving both surgeons and medical oncologists, it should be possible, to translate the antitumour activity of the new first-line regimens into long-term survival benefit for patients with initially unresectable colorectal liver metastases.

## Conclusions

The FOLFIRI regimen, consisting of irinotecan with continuous FA/5-FU over 48 h, given on an outpatient basis was safe and well tolerated in our study. The rate of severe side effects was comparably low with this bi-monthly regimen. As tumor control was achieved in about 75% and downstaging of metastatic disease was possible in some cases, combinations of irinotecan plus continuous FA/5-FU should be further investigated in neoadjuvant protocols for initially unresectable liver metastases.

## List of abbreviations

CRC Colorectal Cancer

FOLFIRI chemotherapeutic regimen consisting of irinotecan combined with continuous FA/5-FU infusions

FA Folinic Acid

5-FU 5-Fluorouracil

HD-5-FU high dose 5-FU

NCI CTC National Cancer Institute Common Toxicity Criteria

CR Complete remission

PR Partial response

SD Stable disease

PD Progressive disease

TTP Time to progression

OS Overall survival

## Author's contributions

MM, SS and AT drafted the manuscript. MM and MH initiated the study. JS, CZ, HH, BA, MS, OK, T, PG and MH were involved in the patient's treatment as well as the documentation of response and side effects.

## Pre-publication history

The pre-publication history for this paper can be accessed here:



## Figures and Tables

**Table 1:** Patient characteristics of all 35 patients with respect to primary tumor side, metastatic sites, number of metastatic sites and previous treatment as well as gender and age.

**Characteristics**	**No.**
**No. Patients**	35
**Gender, male/female**	25/10
**median age, years (range)**	62 (38–75)
**Primary tumor site**	
*Colon*	21
*Rectum*	12
*Ileum*	1
**Metastatic sites**	
*Liver*	26
*Lung*	12
*Lymph nodes*	6
*Local relapse*	3
*other sites*	3
*Peritoneum*	8
**No. of metastatic sites**	
*1*	14
*2*	17
*≥3*	4
**Previous treatment**	
*surgery only*	28
*surgery+radiotherapy+adjuvant chemotherapy*	1
*surgery+adjuvant chemotherapy*	5

**Table 2:** Hematologic toxicity. Number of patients affected with each side effect are listed in the corresponding rows.

**NCI CTC Grade**	**1**	**2**	**3**	**4**
**Leukopenia**	7	1	0	1
**Neutropenia**	4	7	3	0
**Anaemia**	3	1	0	0

**Table 3:** Non-hematologic toxicities. Number of patients affected with each side effect are listed in the corresponding rows.

**NCI CTC Grade**	**1**	**2**	**3**	**4**
**Nausea/Vomiting**	9	8	4	0
**Acute diarrhea**	3	0	2	1
**Delayed diarrhea**	4	4	1	1
**Cholinergic syndrome**	2	0	0	0
**Fever**	3	2	0	0
**Mucositis**	5	0	1	0
**Obstipation**	8	0	0	0
**Asthenia**	2	0	0	0
**Alopezie**	9	1	0	0

**Table 4:** Summary of the response of 35 evaluated patients, divided into CR, PR, SD and PD.

**Response**	No.(%)
Complete Response	4 (11)
Partial Response	7 (20)
Overall Response Rate (CR+PR)	11 (31)
Stable disease	15 (43)
Tumor control rate (CR+PR+SD)	26 (74)
Progressive disease	9 (26)
